# The impact of exercise self-efficacy on college students’ emotion management ability: an analysis of the mediating effects based on exercise behavior and screen media use

**DOI:** 10.3389/fpubh.2024.1456753

**Published:** 2024-10-30

**Authors:** Weidong Zhu, Bo Li, Hu Lou, Fanzheng Mu, Jun Liu

**Affiliations:** College of Sports Science, Nantong University, Nantong, China

**Keywords:** exercise self-efficacy, exercise persistence, physical exercise, screen media use, emotion management ability, mediating effects

## Abstract

**Objective:**

To explore the impact of exercise self-efficacy on college students’ emotion management ability and to analyze the mediating effects of exercise behavior and screen media use between exercise self-efficacy and emotion management ability.

**Methods:**

This study adopted stratified, whole-cluster, and staged sampling methods, using an online questionnaire that included demographic information, exercise self-efficacy, exercise behavior, screen media use, and other relevant aspects, obtaining a total of 12,687 valid questionnaires.

**Results:**

The study indicated a significant positive correlation between exercise self-efficacy and emotion management ability (*r* = 0.349, *p* < 0.01). There was also a positive correlation between physical exercise and emotion management ability (*r* = 0.128, *p* < 0.01). In contrast, smartphone use showed a significant negative correlation with emotion management ability (*r* = −0.102, *p* < 0.01). Additionally, exercise persistence and electronic health literacy presented significant positive correlations with emotion management ability (*r* = 0.370, *p* < 0.01; *r* = 0.502, *p* < 0.01). Chain-mediated effect analysis revealed that exercise self-efficacy positively affected emotion management ability by enhancing physical exercise and reducing smartphone use (95% *CI*: [0.001, 0.002]). Furthermore, exercise self-efficacy positively influenced emotion management ability by enhancing exercise persistence and e-health literacy (95% *CI*: [0.029, 0.042]). These two variables acted as chain mediators, demonstrating the pathways through which exercise self-efficacy affects emotion management ability.

**Conclusion:**

This study deepened the understanding of the interplay between exercise behavior, screen media use, and emotion management ability. It suggested that emotion management ability could be enhanced through strategies of improving exercise persistence, physical exercise, electronic health literacy, and reducing smartphone use while considering differences in gender, academic year, and regional factors in intervention programs.

## Introduction

Emotion management ability was proposed by psychologists Peter Salovey and John D. Mayer. It refers to an individual’s comprehensive ability to recognize, understand, regulate, and express emotions (1). Although emotional intelligence and emotion management ability are related concepts, they are distinct. Emotional intelligence encompasses a broader range of abilities, including recognizing, understanding, using, and managing emotions in various contexts. In contrast, emotion management ability focuses primarily on the regulation and control of emotions, particularly in stressful situations (2). Chinese scholar Wang divides emotional intelligence into four dimensions: perceiving emotions, self-emotion management, managing others’ emotions, and utilizing emotions (3). Individuals with weak emotional intelligence, particularly in managing emotions, are prone to falling into negative emotional states (4). The 2022 Survey Report on the Psychological Health Status of College Students indicates that 21.48% of Chinese college students may be at risk of depression, and 45.28% of college students may be at risk of anxiety (5). Negative emotions such as anxiety and depression can affect the physical and mental health of college students, and in severe cases, even lead to the risk of self-harm and suicide (6). Studies have shown that there is a significant positive correlation between the level of mental health and emotional intelligence, particularly emotion management ability (7). Research has shown that long-term exposure of college students to negative emotions can damage their cognitive functions, with external manifestations such as delayed thinking and decreased willpower (8). People with severe negative emotions can develop psychological issues such as depression, which may lead to suicidal tendencies (9). Students with stronger emotional intelligence, specifically in emotional management, can adjust their emotional state promptly to maintain mental and physical health while facing negative emotions (10). They are also able to effectively cope with stress in life and academics, displaying optimism, a sense of humor, and timely relief of stressful psychological states (11). Currently, emotional intelligence, especially its role in emotion regulation, is a key focus of psychological research at home and abroad, particularly regarding the influencing factors between emotions and behavior.

Building on this understanding of emotional intelligence, it is essential to consider self-efficacy, especially in exercise, as another significant psychological factor influencing students’ well-being. Self-efficacy refers to an individual’s confidence in their ability to perform specific tasks, demonstrating their confidence and autonomy (12). Exercise self-efficacy, as an important component of personal belief, focuses on an individual’s confidence in persisting in fitness activities. It has been recognized as a key factor influencing the psychological development of students, and the enhancement of exercise self-efficacy has been regarded as an important pathway to promote the development of an individual’s physical and mental health (13). College students with high exercise self-efficacy are more likely to make more efforts and persist in various exercises, believing that exercise brings long-term physical and mental health benefits, making it easier for them to derive pleasure from exercising (14). In contrast, college students with low exercise self-efficacy are more likely to substitute pleasurable activities for exercise, and when encountering setbacks in activities, they are more apt to doubt their abilities, leading to a negative cycle of exercise and negative emotions as time goes on (15). Given the close link between college students’ exercise self-efficacy and psychological aspects, academic research has focused on the interaction between exercise and mental health, especially concerning how to improve individuals’ psychological and emotional states by enhancing exercise self-efficacy.

Social cognitive theorists believe self-efficacy plays a special role in emotion management ability, affecting individuals’ thoughts and actions, and enabling them to choose and implement effective emotion management strategies. According to self-efficacy theory, an individual’s confidence level in their ability to accomplish a specific task has a decisive impact on their psychological state and behavioral choices (12). In recent years, much of the research has focused on the effects of different self-efficacies on emotion management ability (16). Specifically, self-efficacies such as academic self-efficacy, social self-efficacy, and exercise self-efficacy have been studied in terms of their unique influences (17, 18). Among these, exercise self-efficacy, which refers to an individual’s belief in their ability to engage in physical activity, has shown a significant impact on emotion management (19). Researchers have found a moderate correlation between self-efficacy and emotion management ability, and self-efficacy can predict the development of emotion management ability (20).

Exercise behavior refers to an individual’s behavior pattern of participating in physical activities, including the type, frequency, intensity, and duration of activities chosen, as well as the degree of continuity and regularity of these activities, mainly involving physical exercise and exercise persistence (21). Exercise persistence represents an individual’s determination and consistency to continue physical activity (22). Previous research has shown that exercise persistence has a positive regulatory effect on psychological emotions, partly due to an improvement in an individual’s self-awareness and self-control of emotion fluctuations (23). Previous studies have also found that physical exercise has a significant positive impact on emotion management ability (24–26).

Screen media use refers to individuals’ interactive behaviors with digital interfaces accessed through screens. Smartphone use and e-health literacy are two key components of screen media use (27, 28). Smartphones combine the functions of computing and mobile phones, and users can engage in gaming, the internet, entertainment, information acquisition, social networking, and communication via smartphone use (29). Media dependency theory suggests that the extent to which an individual relies on a medium depends on the importance of the media in meeting their specific needs (30). The theory proposes a framework for analyzing how people rely on media information to meet their needs and how this media dependency affects individuals’ perceptions, cognition, and behaviors (31). Media dependency theory provides a theoretical basis for analyzing the impact of screen media use on emotion management ability. E-health literacy refers to an individual’s ability to efficiently find, obtain, understand, evaluate, and apply health information via electronic media (32). It is a key factor in the Health Belief Model (33). The Health Belief Model suggests that an individual’s cognition and beliefs about their health are critical determinants of whether they will adopt healthy behaviors (34). Individuals are more likely to take corresponding actions when they perceive themselves at risk or recognize that a certain behavior is beneficial to their health. Studies have found that e-health literacy has a significant positive impact on college student’s mental health, and the potential mechanism for this positive impact may be that high e-health literacy can help students better understand their health status and develop healthy strategies, thereby reducing anxiety and enhancing mental health (35).

**Figure 1:**
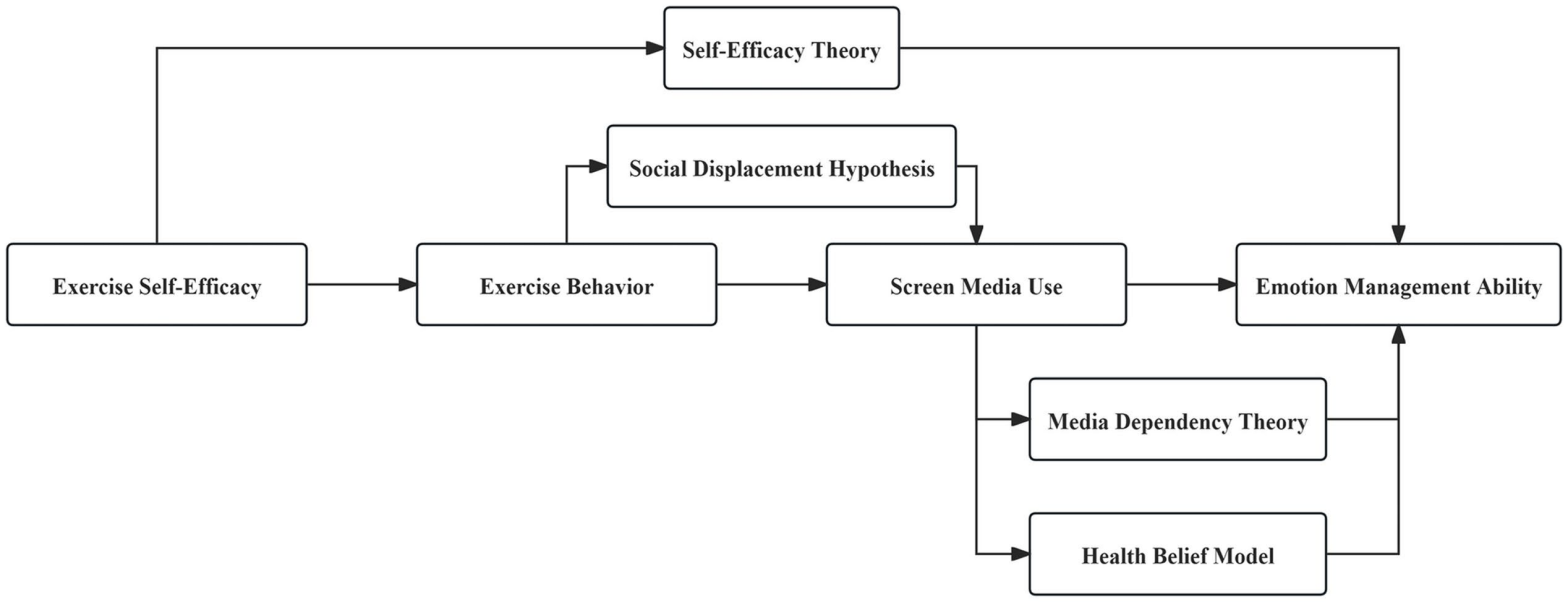
Theoretical model.

This study explores how college students’ exercise self-efficacy affects their emotional management capabilities, incorporating physical activity behavior and screen media usage as mediating variables. The social displacement hypothesis suggests that individuals’ online activities can replace their interactions with others in real life (36). With the development of technology, screen media use occupies an important place in the daily lives of college students, and has become one of the critical factors in altering their lifestyle. According to the results of the 8th Chinese Students’ Physical Fitness and Health Survey, the physical fitness of Chinese college students shows a declining trend (37). Based on the social displacement hypothesis, it is assumed that college students spend too much time on physical exercise, which could correspondingly reduce the frequency of problematic smartphone use (38, 39). The research is based on self-efficacy theory, media dependency theory, the health belief model, and the social displacement hypothesis, to elucidate how self-efficacy influences emotional management through multiple pathways. The theoretical model is shown in Figure 1.

Self-efficacy theory suggests that students’ confidence in their ability to engage in regular physical activities enhances their emotion management ability. Media dependency theory explains how smartphone usage can affect emotion management ability, while the health belief model elucidates how e-health literacy can help students better manage emotions. The social displacement hypothesis discusses how physical exercise can reduce screen media usage, ultimately impacting emotion management. By integrating these theoretical perspectives, this study aims to clarify the specific mechanisms by which exercise self-efficacy impacts emotional management capabilities, particularly the mediating roles of physical activity behavior and screen media usage.

**Figure 2:**
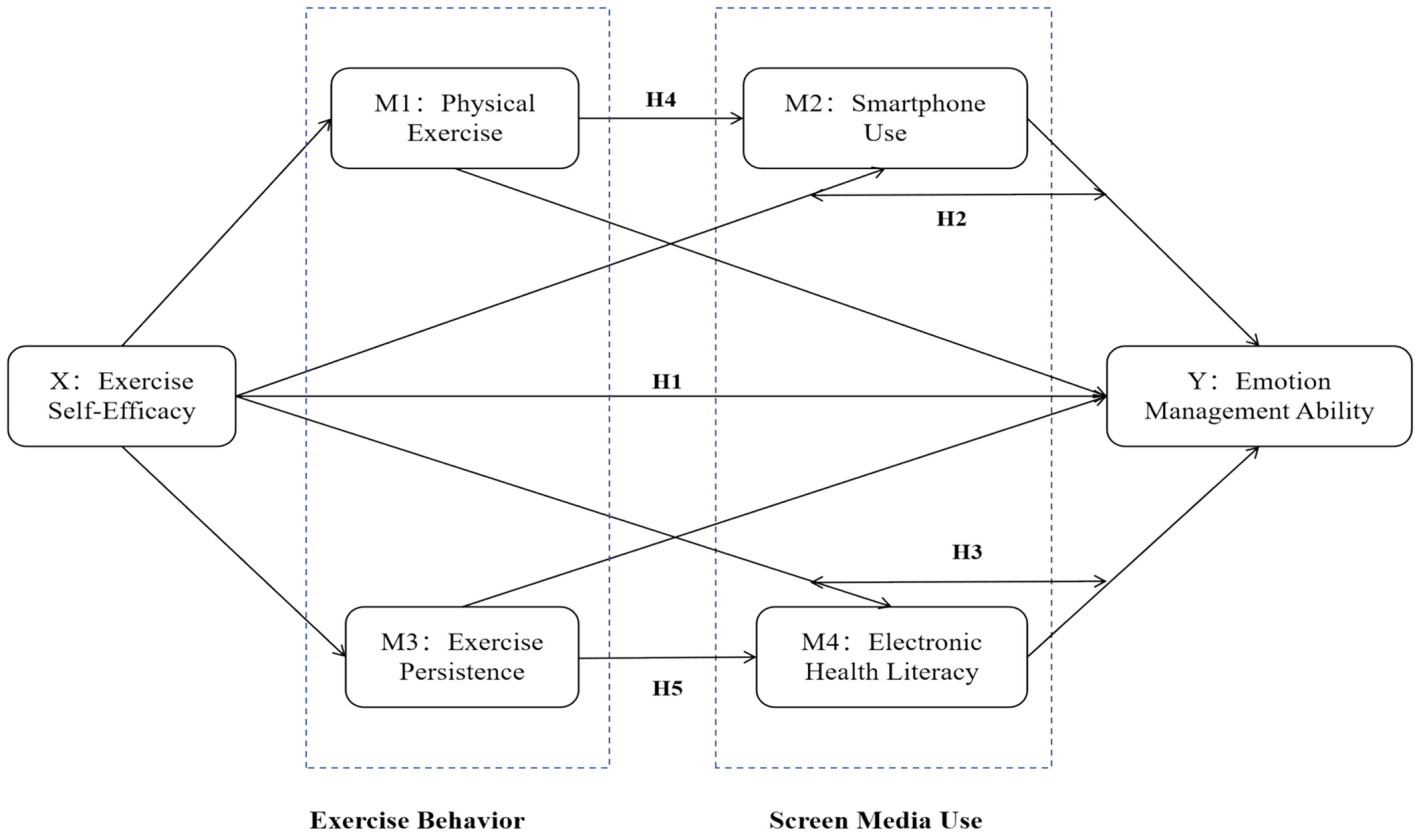
Hypothetical model diagram.

In choosing the specific chains of mediation models, this study considered the direct relevance and significant impact of these factors as highlighted by the integrated theories. The main hypotheses are as follows, and the hypothetical model diagram is shown in Figure 2.

*H1: College students’ exercise self-efficacy has a positive predictive effect on emotion management ability.*

*H2: College students’ smartphone use mediates the relationship between exercise self-efficacy and emotion management ability.*

*H3: College students’ e-health literacy mediates the relationship between exercise self-efficacy and emotion management ability.*

*H4: There is a chain mediating effect of physical exercise and smartphone use between college students’ exercise self-efficacy and their emotion management ability.*

*H5: There is a chain mediating effect of persistence in exercise and e-health literacy between college students’ exercise self-efficacy and their emotion management ability.*

## Methods

### Participants

This study adopted a stratified random cluster sampling method, with data collected in September 2022. The participants were full-time Nanjing Xiaozhuang University, Shangqiu University, and Heihe University College students.

#### Determination of sampling sites

To ensure diversity in terms of geographical and socioeconomic conditions, three sampling sites were selected within mainland China, located in the East China region, North China region, and Central China region.

#### Selection of sampling units

The sampling units were selected based on three key criteria. First, only higher education institutions registered with the Ministry of Education, including both regular and vocational/technical colleges, were eligible to participate. Second, these units needed to meet specific sampling criteria, including student age, the number of enrolled students, and the distribution of students across different grades. Lastly, participating institutions had to designate responsible individuals for questionnaire distribution and demonstrate a willingness to engage in long-term monitoring. The survey was conducted after students had returned to campus for the fall semester.

#### Quality control

Data cleaning rules: Data cleaning was conducted to ensure the external validity of the analyzed data. In the data preprocessing stage, logical errors, omissions, inaccuracies, and indiscernible responses were identified and either retested or excluded to ensure data accuracy and validity. The following rules were applied for the inclusion of valid questionnaires: (1) questionnaires with unidentifiable school full names were deleted, resulting in the exclusion of 97 questionnaires. (2) questionnaires with codes indicating complete consistency for at least 21 consecutive entries were deleted, resulting in the exclusion of 624 questionnaires. (3) questionnaires with completion times falling within the ranges of (0, 0.5%] and (99.5%, 100%], given that the average completion time was 6 min and 12 s, were excluded. Finally, a total of 12,687 valid questionnaires were included in the analysis. Out of the total 13,408 questionnaires distributed, 12,687 were deemed valid after screening, resulting in a response rate of approximately 94.6%.

#### Stratification and sample size

The sample population was divided into two primary groups based on gender (male and female) and further stratified into eight categories according to grade level. Each category required a minimum sample size of 45 individuals. The total sample size per region was set at 360 participants, aiming for a combined total of 1,080 participants across the three regions. The questionnaire was electronically administered using the Questionnaire Star platform in late September 2022, ultimately collecting 12,687 valid responses. The distribution of the sample is shown in Table 1.

**Table 1:** Sample characteristics table.

Variable		*n*	%
Gender	Male	4,595	36.2
	Female	8,092	63.8
Grade	Freshmen	3,510	27.7
	Sophomores	3,505	27.6
	Juniors	4,127	32.5
	Seniors	1,545	12.2
Region	Eastern	3,505	27.6
	Central	7,722	60.9
	Northern	1,460	11.5
Total		12,687	100

Questionnaires were completed using the Questionnaire Star software (version 2.0.96) for the various scales and informed consent forms. The questionnaire software (version 2.0.96) is a tool specifically designed for survey research, supporting the electronic management and distribution of various scales and questionnaires. This software not only efficiently handles the completion of informed consent forms but also offers a user-friendly interface that allows respondents to intuitively complete the surveys. Through this software, the accuracy and completeness of data can be ensured, while significantly enhancing the efficiency of the survey process. Before distributing the questionnaires, 15 surveyors were trained to assist with the distribution. The 15 surveyors are counselors from various majors at Nanjing Xiaozhuang University, Shangqiu University, and Heihe University College. The final number of people included in the data analysis was 12,687. The research plan was approved by the Ethics Committee of Nantong University [2022(70)]. Informed consent was obtained from all subjects included in the study. All methods of this study were performed following relevant guidelines and regulations. The formula for calculating the minimum sample size is given by Equation 1, with the Type I error (*α*) set to 0.05, the allowable error (*δ*) set to 0.01, and the sampling rate (*ρ*) set to 0.05 (40). The total number of students at Nanjing Xiaozhuang University, Shangqiu University, and Heihe University College totaled 53,614 (data updated in August 2023). The finite total number *N* is set at 75% of the total number of students, which is 40,211. The minimum sample size required for this study was calculated to be 1,746.


(1) $$n=\frac{{\left(\frac{Z_{\alpha }}{\delta }\right)}^{2}*p*\left(1-p\right)}{1+\left[{\left(\frac{Z_{\alpha }}{\delta }\right)}^{2}*p*\left(1-p\right)\right]/N}$$

### Measurement

#### Exercise self-efficacy

The exercise self-efficacy of university students was assessed using the Physical Activity Self-efficacy Scale (PASS), developed by Jiang (41). The PASS consists of two dimensions namely situational motivation and subjective support, with a total of 10 items, scored on a Likert 5-point scale ranging from “1 = strongly disagree” to “5 = strongly agree.” Higher item scores indicate a higher sense of exercise self-efficacy. The cumulative variance contribution rate of the two factors is 67.782%. The results of the confirmatory factor analysis show that the two-factor model fits well (*χ^2^/df* = 2.688, *GFI* = 0.958, *AGFI* = 0.932, *NFI* = 0.970, *CF*I = 0.981, *IFI* = 0.975, *RMSEA* = 0.066, *RMR* = 0.023), and the scale’s Cronbach’s α coefficient, split-half reliability, and test-retest reliability are 0.908, 0.819, and 0.531, respectively (41).

#### Exercise behavior

##### Physical activity level

The Physical Activity Rating Scale-3 (PARS-3) developed by the Japanese scholars, Tokunaga, and Hashimoto, and revised by the Chinese scholar, Liang (42), was used. The scale comprises 3 items, each rated on a 5-point scale, with total scores ranging from 0 to 100. Higher scores indicate greater physical activity. The scoring criteria are low activity (≤ 19 points), medium activity (20–42 points), and high activity (≥ 43 points). The calculation formula for physical exercise is shown as Equation 2. Studies have shown that the scale has an internal consistency reliability of 0.856 and a test–retest reliability of 0.820 (42).


(2) $$\begin{array}{l} \mathrm{Physical}\;\mathrm{Exercise}=\mathrm{frequency}\;\mathrm{of}\;\mathrm{physical}\;\mathrm{exercise}\\ \;\times \mathrm{intensity}\;\mathrm{of}\;\mathrm{physical}\;\mathrm{exercise}\\ \;\times \left(\mathrm{duration}\;\mathrm{of}\;\mathrm{physical}\;\mathrm{exercise}-1\right) \end{array}$$

##### Exercise persistence

The Exercise Persistence Scale (EPS), developed by Gu of Fujian Normal University (43), was used. EPS consists of three dimensions, exercise behavior, effort engagement, and emotional experience, totaling 14 items, with a 5-level rating system. The higher the score, the greater the persistence in exercise. The reliability of the EPS is 0.947, with fit indices of *χ^2^/df* = 2.896, *CFI* = 0.945, *GFI* = 0.901, and *RESEA* = 0.069, indicating that the scale has good reliability and validity and can be used as a general tool in research (43). In research targeting the university student population, Cronbach’s *α* is 0.892, and test–retest reliability is 0.874 (44).

#### Screen media usage

##### Smartphone use

The degree of smartphone usage among college students was measured using the Mobile Phone Addiction Tendency Scale (MPATS) developed by the Chinese scholar Xiong (45). MPATS encompasses four dimensions, which are withdrawal symptoms, salience behavior, social comfort, and mood changes, and it comprises a total of 16 questions. Each question is scored using the Likert five-point scale, starting from “completely inconsistent” to “completely consistent,” with scores ranging from 1 to 5 points. The highest score is 80 points and the lowest is 16 points. The higher the score, the greater the tendency for problematic mobile phone use. The lower the score, the lower the tendency for problematic mobile phone use. The Factor loadings of the four dimensions ranged from 0.51 to 0.79, and the variance explanation rate was 54.3%. The results of confirmatory factor analysis demonstrated the applicability of the four-factor model, with fit indices of *χ^2^/df* = 2.92, *RMSEA* = 0.07, *CFI* = 0.96, *NFI* = 0.94, *IFI* = 0.96, *RFI* = 0.93. The Cronbach coefficient of the total scale was 0.83, the coefficients for the four factors were between 0.55 and 0.80, and the scale’s test–retest reliability was 0.91, with re-test reliability values for the four factors ranging from 0.75 to 0.85 (45). In research targeting the university student population, Cronbach’s *α* is 0.910, and test–retest reliability is 0.690 (46).

##### E-health literacy

The e-Health Literacy Scale (eHEALS) was developed by Norman et al. and was translated into simplified Chinese by Guo (47). The scale consists of 8 items and includes the application ability test of online health information and services (items 1–5), the judgment ability test (items 6–7), and the decision-making ability test (item 8). Each item adopts the 5-point Likert scale, from “very inconsistent” to “very consistent,” scoring 1–5 points respectively, with total scores ranging from 8 to 40. Higher scores indicate higher levels of e-health literacy. In this study, Cronbach’s *α* coefficient of the scale was 0.913, the KMO value was 0.875, and Bartlett’s test of sphericity value was 544.000 (47). In research targeting the university student population, Cronbach’s *α* is 0.915 (48).

##### Emotion intelligence scale

The Emotion Intelligence Scale (EIS), translated by the Chinese scholar Wang (3), was used to measure the emotion management ability of university students. The EIS consists of 33 items, encompassing 4 subscales. This study used the emotion management ability subscale, consisting of items 2, 6, 7, 10, 12, 14, 21, and 28 from the EIS. There are 8 items in total, scored on a 5-point Likert scale from 1 (“very inconsistent”) to 5 (“very consistent”). The higher the score, the better the emotion management ability. The Cronbach’s alpha coefficient for the total scale was 0.840, and the criterion predictive validity was 0.320 (*p* < 0.01) (49).

### Statistical analysis

The data processing in this study was conducted using SPSS 27.0 and Excel software. The process is divided into the following steps:

1. Pre-processing the data obtained from the Questionnaire Star via Excel software, including retaking or deleting missing or problematic data.
2. Performing tests for common method bias to avoid issues related to this.
3. Analyzing the collected student data for the situation of emotion management ability analyze differences in emotion management ability among students of different genders grades, and regions, with η^2^ values ranging from 0 to 1. According to Cohen’s standards, a value of 0.01 represents a small effect means that the difference or relationship being measured has a minimal impact., 0.06 is a medium effect indicating a moderate effect., and 0.14 is a large effect representing a strong or substantial effect (50). An F-test was used to assess if there were significant differences in variances between multiple groups. This test is crucial for determining if groups show significantly different variance patterns.
4. Using Pearson correlation analysis to test the relationships between university students’ exercise self-efficacy (situational motivation, subjective support), physical exercise, exercise persistence (exercise behavior, effort engagement, emotion experience), smartphone use (withdrawal symptoms, salience behavior, social comfort, and mood changes), e-health literacy (application ability, judgment ability, decision-making ability), and emotion management ability (51).
5. Using regression analysis to test for mediation effects and the Process plugin to conduct mediation effect analysis (52). By repeatedly resampling the original data, with the number of resamples set to 5,000, the indirect effects of each path and their confidence intervals are calculated, thus providing a more robust evaluation of the significance of the chain mediation effects.

## Results

### Test for common method Bias

In this study, individually completed subjective scales were used for data collection. To avoid common method bias, after obtaining the data, Harman’s single-factor test was used to conduct an exploratory factor analysis on all questionnaire items for the variables of exercise self-efficacy, physical exercise, smartphone usage, exercise persistence, e-health literacy, and emotion management ability. The results showed that we extracted 8 main components with eigenvalues greater than 1, and the maximum factor explained 28.969% of the variance, which is less than the commonly set threshold of 40%. Therefore, we can conclude that there is no common method bias in this study (53).

### Descriptive analysis

From Table 2, the average score of college students’ exercise self-efficacy was 37.140 ± 8.905; male students scored 38.950 ± 8.618, while female students scored 36.110 ± 8.903. The scores were similar across grades (*p* = 0.128, *η^2^* < 0.001). By region (*p* < 0.001, *η^2^* = 0.002), the eastern (37.623 ± 8.942) and northern (37.580 ± 10.050) regions had higher scores than the central (36.836 ± 8.643) region. For emotion management ability, college students scored 29.380 ± 5.327, with no significant gender differences (*p* = 0.003, *η^2^* = 0.001). The total smartphone usage score was 39.310 ± 15.684, with gender differences being insignificant (*p* = 0.011, *η^2^* = 0.001), and sophomores scoring higher (41.070 ± 15.966) than other grades (*p* < 0.001, *η^2^* = 0.006). Regionally, scores from the eastern (13.575 ± 18.010) region were higher than those from the central (11.434 ± 16.356) and northern (10.769 ± 17.160) regions (*p* < 0.001, *η^2^* = 0.005). The total exercise persistence score was 48.870 ± 11.358, with males scoring higher (51.620 ± 11.870) than females (46.310 ± 10.750), with a significant difference (*p* < 0.001, *η^2^* = 0.033). For e-health literacy, students scored 28.170 ± 8.155, showing no significant gender differences (*p* = 0.002, *η^2^* = 0.001). The physical exercise score was 11.950 ± 16.951, with males scoring significantly higher (18.770 ± 22.289) than females (8.080 ± 11.269) (*p* < 0.001, *η^2^* = 0.092).

**Table 2:** Overview of descriptive analysis results.

			Exercise self-efficacy	Emotion management ability	Exercise behavior		Screen media use	
					Physical Exercise	Exercise Persistence	Smartphone Use	Electronic Health Literacy
Gender		M	37.140	29.380	11.950	48.870	39.310	28.170
		sd	8.905	5.327	16.951	11.358	15.684	8.155
	Male	M	38.950	29.190	18.770	51.620	39.780	27.860
		sd	8.618	5.776	22.289	11.870	17.082	8.722
	Female	M	36.110	29.490	8.080	47.310	39.040	28.340
		sd	8.903	5.052	11.269	10.750	14.825	7.810
	*F*		304.518	9.050	1284.782	435.213	6.519	9.944
	*p*		<0.001	0.003	<0.001	<0.001	0.011	0.002
	*η*^2^		0.023	0.001	0.092	0.033	0.001	0.001
Grade	Freshman	M	37.130	30.010	11.360	48.570	37.940	28.760
		sd	8.454	4.771	14.928	10.459	13.949	7.478
	Sophomores	M	37.260	29.420	12.070	49.080	41.070	28.230
		sd	8.920	5.180	17.285	11.342	15.966	8.034
	Juniors	M	36.910	28.940	11.980	48.710	39.010	27.660
		sd	9.183	5.608	17.693	11.896	16.211	8.511
	Seniors	M	37.480	29.010	12.950	49.510	39.190	28.020
		sd	9.112	5.910	18.383	11.851	16.928	8.815
	*F*		1.898	28.473	3.259	3.091	24.296	11.708
	*p*		0.128	<0.001	0.021	0.026	<0.001	<0.001
	*η*^2^		<0.001	0.007	0.001	0.001	0.006	0.003
Region	Eastern	M	37.623	29.959	13.575	49.699	13.575	49.699
		sd	8.942	5.068	18.010	11.412	18.010	11.412
	Central	M	36.836	29.088	11.434	48.263	11.434	48.263
		sd	8.643	5.255	16.356	10.956	16.356	10.956
	Northern	M	37.580	29.514	10.769	50.105	10.769	50.105
		sd	10.050	6.134	17.160	12.985	17.160	12.985
	*F*		11.472	32.964	23.311	29.119	60.454	66.710
	*p*		<0.001	<0.001	<0.001	<0.001	<0.001	<0.001
	*η* ^2^		0.002	0.005	0.004	0.005	0.009	0.010

### Correlation analysis

From Table 3, it is apparent that there is a significant positive correlation between exercise self-efficacy and its, situational motivation, subjective support, and emotion management ability, with correlation coefficients ranging between 0.326 and 0.357. Physical exercise and its sub-dimension, exercise level, show a significant positive correlation with emotion management ability, with correlation coefficients ranging between 0.101 and 0.128. Smartphone usage and its sub-dimensions, withdrawal symptoms, prominent behavior, social soothing, and mood change demonstrate a significant negative correlation with emotion management ability, with correlation coefficients ranging from −0.146 to −0.053. The total score of exercise persistence and its sub-dimensions, exercise behavior, effort engagement, emotion experience, and emotion management ability show a significant positive correlation, with correlation coefficients between 0.269 and 0.386. E-health literacy and its sub-dimensions, application ability, judgment ability, and decision-making ability are significantly positively correlated with emotion management ability, with correlation coefficients ranging between 0.480 and 0.502. Exercise self-efficacy is significantly positively correlated with physical exercise, exercise persistence, and e-health literacy, but is significantly negatively correlated with smartphone usage. Physical exercise is significantly negatively correlated with smartphone usage. Exercise persistence shows a significant positive correlation with e-health literacy.

**Table 3:** Correlation analysis between various variables and emotion management ability.

	1	1-1	1-2	2	3-1	3-2	4	4-1	4-2	4-3	5	5-1	5-2	5-3	5-4
1	1														
1-1	0.981^**^	1													
1-2	0.956^**^	0.881^**^	1												
2	0.349^**^	0.326^**^	0.357^**^	1											
3-1	0.292^**^	0.282^**^	0.286^**^	0.128^**^	1										
3-2	0.253^**^	0.245^**^	0.247^**^	0.101^**^	0.909^**^	1									
4	0.767^**^	0.749^**^	0.738^**^	0.370^**^	0.362^**^	0.321^**^	1								
4-1	0.695^**^	0.690^**^	0.651^**^	0.269^**^	0.380^**^	0.343^**^	0.908^**^	1							
4-2	0.731^**^	0.710^**^	0.710^**^	0.358^**^	0.337^**^	0.297^**^	0.955^**^	0.829^**^	1						
4-3	0.666^**^	0.643^**^	0.652^**^	0.386^**^	0.267^**^	0.231^**^	0.863^**^	0.637^**^	0.753^**^	1					
5	−0.069^**^	−0.055^**^	−0.085^**^	−0.102^**^	−0.052^**^	−0.032^**^	−0.020^*^	0.013	−0.028^**^	−0.040^**^	1				
5-1	−0.071^**^	−0.061^**^	−0.079^**^	−0.053^**^	−0.049^**^	−0.032^**^	−0.019^*^	−0.009	−0.026^**^	−0.017^*^	0.958^**^	1			
5-2	−0.027^**^	−0.006	−0.055^**^	−0.146^**^	−0.034^**^	−0.014	0.014	0.071^**^	0.002	−0.038^**^	0.923^**^	0.839^**^	1		
5-3	−0.106^**^	−0.095^**^	−0.115^**^	−0.100^**^	−0.080^**^	−0.059^**^	−0.068^**^	−0.042^**^	−0.071^**^	−0.072^**^	0.885^**^	0.804^**^	0.749^**^	1	
5-4	−0.052^**^	−0.039^**^	−0.068^**^	−0.112^**^	−0.038^**^	−0.020^*^	−0.008	0.031^**^	−0.015	−0.040^**^	0.928^**^	0.852^**^	0.851^**^	0.768^**^	1
6	0.181^**^	0.166^**^	0.190^**^	0.502^**^	0.070^**^	0.063^**^	0.202^**^	0.133^**^	0.197^**^	0.225^**^	0.195^**^	0.225^**^	0.121^**^	0.183^**^	0.162^**^
6-1	0.165^**^	0.150^**^	0.175^**^	0.480^**^	0.062^**^	0.055^**^	0.184^**^	0.116^**^	0.181^**^	0.211^**^	0.200^**^	0.231^**^	0.124^**^	0.192^**^	0.164^**^
6-2	0.189^**^	0.175^**^	0.196^**^	0.499^**^	0.077^**^	0.070^**^	0.211^**^	0.147^**^	0.205^**^	0.227^**^	0.170^**^	0.198^**^	0.105^**^	0.155^**^	0.145^**^
6-3	0.197^**^	0.183^**^	0.203^**^	0.488^**^	0.078^**^	0.071^**^	0.216^**^	0.154^**^	0.209^**^	0.229^**^	0.168^**^	0.194^**^	0.107^**^	0.148^**^	0.144^**^

1 = Exercise Self-efficacy, 1-1 = Situational Motivation, 1-2 = Subjective Support, 2 = Emotion Management Ability, 3-1 = Physical Exercise, 3-2 = Physical Exercise Level, 4 = Exercise Persistence, 4-1 = Exercise Behavior, 4-2 = Effort Investment, 4-3 = Emotion Experience, 5 = Smartphone Usage, 5-1 = Withdrawal Symptoms, 5-2 = Prominent Behavior, 5-3 = Social Soothing, 5-4 = Mood Modification, 6 = Electronic Health Literacy, 6-1 = Application Ability, 6-2 = Judgment Ability, 6-3 = Decision-making Ability.

** The correlation is significant at the 0.01 level.

* The correlation is significant at the 0.05 level.

### Multiple stepwise regression analysis

**Figure 3:**
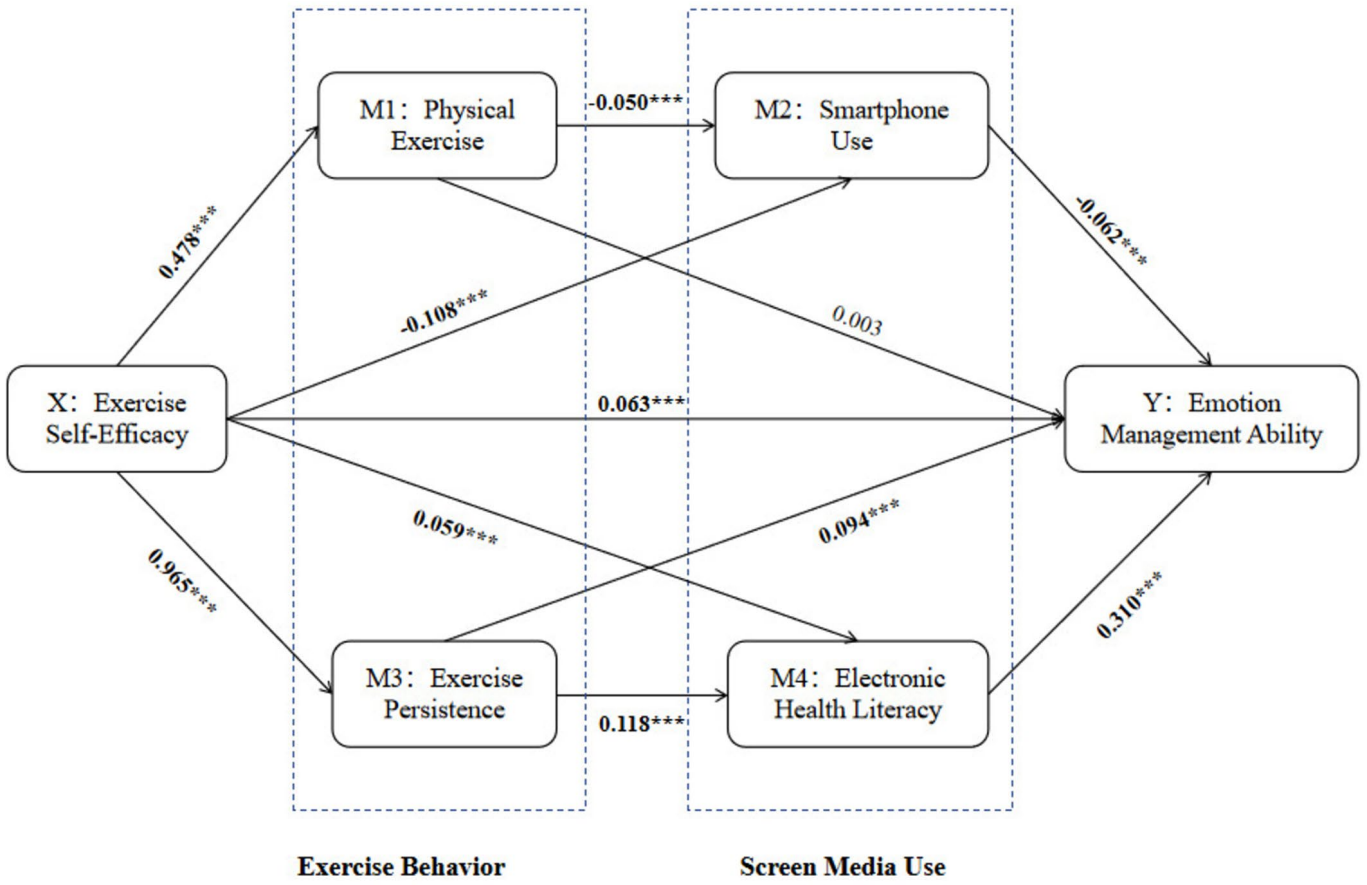
Path analysis diagram.

Using SPSS 27.0 and applying model 82 in the PROCESS tool to test the chain mediation model, and after excluding control variables such as gender and school year, the results were shown in Figure 3 and Table 4 (52). It can be seen that in Model 3, the explanatory power of exercise self-efficacy, amount of physical exercise, smartphone usage, exercise persistence, and e-health literacy on emotion management ability is 37.3%. The results of the variance analysis indicated *F* = 1482.464, with a *p*-value of less than 0.001, demonstrating the suitability of the model. The standardized coefficients for exercise self-efficacy, exercise persistence, and e-health literacy are *β* = 0.105, 0.201, and 0.474, respectively, with *p*-values less than 0.001, indicating that a significant positive impact on emotion management ability.

**Table 4:** Regression analysis of influencing factors with control variables.

Variables	Model 1		Model 2		Model 3	
	*β*	*t*	*β*	*t*	*β*	*t*
Gender	0.024	2.700			0.06	8.128***
Grade	−0.068	−7.365***			−0.052	−7.127***
Region	−0.025	−2.722			−0.003	−0.453
Exercise Self-efficacy			0.104	9.412***	0.105	9.545***
Physical Exercise			−0.017	−2.176	0.001	0.142
Exercise Persistence			0.195	17.132***	0.201	17.752***
Smartphone Usage			−0.186	−25.59***	−0.181	−24.956***
Electronic Health Literacy			0.482	65.256***	0.474	64.156***
Adjusted R-squared	0.007		0.367		0.373	
*F*-value	29.619***		1470.788***		1482.464***	

***Represents *p* < 0.001, **represents *p* < 0.01, *represents *p* < 0.05.

### Test of mediating effects

According to Table 5, the 95% confidence interval (*CI*) for the mediation effect of physical exercise between exercise self-efficacy and emotion management ability is [−0.003, 0.003], with the confidence interval including 0. Therefore, in this study, there is no mediating effect of physical exercise between exercise self-efficacy and emotion management ability. The mediation effects of smartphone usage and exercise persistence between exercise self-efficacy and emotion management ability are, respectively, (0.007, 95% *CI*: [0.004, 0.009]) and (0.091, 95% *CI*: [0.080, 0.103]), with confidence intervals not including 0, indicating the presence of mediation effects between smartphone usage and exercise persistence. Meanwhile, the 95% CI for the joint mediation of physical exercise and smartphone usage between exercise self-efficacy and emotion management ability is [0.001, 0.002], with an effect size of 0.002, suggesting a mediating effect. The 95% CI for the mediating effect of exercise persistence and e-health literacy between exercise self-efficacy and emotion management ability is [0.029, 0.042], with an effect size of 0.035, confirming a mediating effect. Therefore, physical exercise and smartphone usage as well as exercise persistence and e-health literacy, respectively, play a chain-mediated role in exercise self-efficacy and emotion management ability.

**Table 5:** Analysis of the mediating effects of the mediator variables between exercise self-efficacy and emotion management ability.

	Effect	BootSE	LLCI	ULCI	Proportion of effect
Total effect	0.216	0.005	0.206	0.226	
Direct effect	0.063	0.007	0.050	0.076	29.2%
Indirect	0.153	0.006	0.141	0.165	70.8%
Exercise self-efficacy → Physical exercise → Emotion management ability	<0.001	0.001	−0.003	0.003	0
Exercise self-efficacy → Smartphone use → Emotion management ability	0.007	0.001	0.004	0.009	3.1%
Exercise self-efficacy → Exercise Persistence → Emotion management ability	0.091	0.006	0.080	0.103	42.2%
Exercise self-efficacy → Electronic Health Literacy → Emotion management ability	0.018	0.004	0.010	0.027	8.5%
Exercise self-efficacy → Physical exercise → Smartphone use → Emotion management ability	0.002	<0.001	0.001	0.002	0.7%
Exercise self-efficacy → Exercise Persistence → Electronic Health Literacy → Emotion management ability	0.035	0.003	0.029	0.042	16.3%

LLCI, lower-level confidence interval; ULCI, upper-level confidence interval.

## Discussion

This study aims to explore the relationship between exercise behavior and emotion management ability among college students in a higher education environment and further reveal the role of screen media usage in the connection between exercise behavior and emotion management ability. The study found that physical exercise and smartphone use formed a chain-mediated reaction between exercise self-efficacy and emotion management ability, with exercise persistence and e-health literacy also showing a chain-mediated effect. A key insight from this study is that while exercise behavior was expected to mediate this relationship, it did not, suggesting that its role is more complex than previously understood. This study integrates multiple theories—self-efficacy theory, media dependency theory, health belief model, and social displacement hypothesis—to elucidate the relationship between college students’ exercise self-efficacy and emotion management ability. The findings reveal that exercise behavior, contrary to expectations, does not directly mediate this relationship. Instead, screen media usage and other mediators play a significant role, demonstrating that emotional health is shaped by a multifaceted interaction of lifestyle factors. This contrasts with previous research and suggests a more complex role for physical exercise in emotional regulation, potentially moderated by exercise intensity and psychological factors.

The important finding of this study is that physical exercise did not show a mediating effect between exercise self-efficacy and emotion management ability, meaning that exercise self-efficacy did not influence emotion management ability through physical exercise. This is inconsistent with previous studies (54, 55). The study suggests that the lack of mediation effect between physical exercise and exercise self-efficacy and emotion management ability may be due to the psychological stress and anxiety induced by high-intensity physical exercise. Previous research has found that moderate to low-intensity physical exercise has a significant improvement effect on negative emotions, whereas high-intensity exercise may increase negative emotions in individuals (56). However, adding smartphone usage as a mediating variable established a chain mediating path between exercise self-efficacy and emotion management ability. The mediation effect of smartphone usage also confirms it as a potential factor, likely because exercise self-efficacy encourages more engagement in physical exercise and consequently less time spent on smartphone use, ultimately reducing dependence on the device (57), and thereby enhancing emotion management ability (58). Therefore, the interaction between exercise and smartphone usage underscores a multidimensional pathway in which lifestyle factors influence emotional health. Hence, when designing intervention plans to improve emotion management ability could consider combining strategies to enhance physical exercise and control smartphone use.

From the demographic results of each variable, there is a significant difference in emotion management ability across genders and grades. Emotion management ability showed a decreasing trend with increasing grade level. This decreasing trend could be because as the grade level increases, college students begin to think more about employment after graduation. Stress and anxiety about finding a job, career development worries, and work-related expectations can negatively influence emotion management ability (59). The significant gender difference aligns with previous studies, suggesting that females exhibit better emotion management ability than males, possibly related to differences in hormonal secretions between genders (60). E-health literacy did not show significant gender differences, which is different from previous studies (61), potentially because the sample was drawn from college students who generally receive comparable levels of education, health information, and electronic resources in a university setting. Higher education provides an equal platform for information acquisition, helping to reduce gender differences in e-health literacy caused by educational disparities (62). Similarly, smartphone usage did not present significant gender differences, in line with previous findings (63), possibly due to increased usage during the quarantine period of the epidemic. Exercise persistence, physical exercise, and exercise self-efficacy showed significant gender differences but not grade-level differences, which is consistent with earlier research (64). Traditional social-cultural factors, gender role concepts, and physiological differences can lead to differences in physical exercise among different genders (65, 66).

This study finds that exercise self-efficacy significantly positively predicts emotion management ability (*β* = 0.063), thereby validating hypothesis H1. High exercise self-efficacy was found to be positively correlated with better emotion management ability, suggesting that individuals who are confident in their ability to exercise are also more likely to believe they can effectively cope with emotional challenges (67). The positive correlation between exercise self-efficacy and emotion management ability may stem from increased intrinsic motivation, successful exercise experiences, and enhanced adaptability and problem-solving skills, leading to more stable emotion regulation (68).

The study shows a mediating effect of smartphone usage between physical exercise self-efficacy and emotion management ability, accounting for 3.1% of the effect, supporting hypothesis H2. High exercise self-efficacy may lead individuals to believe they can effectively manage and adjust their health behaviors, including reducing excessive smartphone usage (69). This reduced smartphone usage can free up more time and energy for physical exercise activities, ultimately elevating emotion management ability (70). The reduction in smartphone usage aligns with the social displacement hypothesis.

The study also reveals a mediating effect of e-health literacy between exercise self-efficacy and emotion management ability, comprising 8.5% of the effect, validating hypothesis H3. When individuals are confident and believe they can successfully engage in physical exercise, they may be more proactive in searching for, understanding, and utilizing health-related electronic information (71). By managing health efficiently through reliable information, healthy behaviors may facilitate an improvement in emotion management ability (72). This aligns with the constructs of the Health Belief Model.

Additionally, the study shows a sequential mediation effect of physical exercise and smartphone use between exercise self-efficacy and emotion management ability, accounting for 0.7% of the effect, establishing hypothesis H4. Exercise self-efficacy may increase the frequency and intensity of an individual’s participation in physical exercise, as individuals perceive themselves to be more able to engage in regular exercise (73). The increase in individual participation in physical exercise could then impact smartphone usage, potentially by reducing leisure time spent on the device., enhance emotion management ability.

Finally, the study shows that exercise persistence and e-health literacy have a chain mediation effect between exercise self-efficacy and emotion management ability, accounting for 16.3% of the effect, confirming hypothesis H5. previous research indicated that high exercise self-efficacy may enhance exercise persistence, as individuals feel more capable of sustaining workouts (74). Persistent exercise behavior can lead to increased searching for, understanding, and application of health-related electronic information, likely due to the individual’s desire to better manage their health or optimize their exercise plan Improved e-health literacy enables individuals to more effectively access and utilize health information, including strategies that may aid in better regulation and management of emotions, thereby enhancing emotion management ability (75).

To explore the mediation effects of exercise behavior and screen media usage between exercise self-efficacy and emotion management ability, this study utilizes process model 82 to validate the theoretical framework (52). The analysis results indicate a mediation effect of screen media usage between exercise self-efficacy and emotion management ability. Exercise self-efficacy exhibits a significant positive influence on individual exercise behavior and emotion management. The mediation effect of screen media usage suggests rational use can serve as a tool for information acquisition and skill enhancement, with excessive reliance possibly leading to adverse effects. Consequently, the study advocates for higher education environments to support and encourage sensible screen media usage and the development of exercise self-efficacy. The mediation effect of screen media usage is multifaceted, encompassing both time management and emotional aspects. While rational use can serve as a tool for information acquisition and skill enhancement, excessive reliance may lead to adverse effects on both time allocation and emotional well-being. It’s important to note that the “Smartphone use” tool employed in this study not only measures the time spent on devices but also captures emotional dependence and psychological attachment to smartphones.

Furthermore, this study also considers the reverse causal perspective, exploring how good emotion management skills can in turn enhance exercise self-efficacy, as well as positively influence physical exercise behavior and the use of health information. If individuals can effectively manage their emotions, this adept emotional control can not only boost their confidence and motivation to engage in sports activities but also directly enhance their confidence in their physical abilities, thus, exercising self-efficacy. Further, efficient emotional management skills may encourage individuals to use screen media more purposefully and logically, utilizing it as a tool to acquire health information and workout inspiration, rather than merely for entertainment, thereby freeing up more time and energy for physical exercise. Moreover, better emotional regulation encourages individuals to actively search for and apply health information to support health decisions and exercise planning, and this proactive attitude is expected to further nurture their exercise self-efficacy. In this reverse action mechanism, the mutually reinforcing relationship between emotion management abilities and exercise self-efficacy unveils new thoughts for establishing more comprehensive intervention measures. These measures should include not only direct interventions to enhance exercise self-efficacy but also training in emotional management skills and reasonable guidance on the use of screen media, aiming to promote an overall improvement in individuals’ emotional and physical health levels.

### Limitation

The limitations of this study include: First, the sample collection was overly concentrated, as only schools from the eastern, central, and western regions of China were sampled, which may increase the likelihood of selection bias. Second, there was a reliance on recall-based questionnaires without instrumental testing. Third, significant endogeneity issues may exist in the analytical framework, including potential reverse causality. This complicates the interpretation of the directionality of causation between variables, potentially leading to misinterpretations of the analysis. Even where statistical correlations exist, they should be interpreted with caution, as the exact causal relationships remain unclear.

## Conclusion

The positive association between exercise self-efficacy and emotion regulation ability is influenced by multiple mediating factors, including physical exercise, reduced use of smartphones, exercise persistence, and enhanced e-health literacy. This study’s key finding is that physical exercise did not directly mediate the relationship between exercise self-efficacy and emotion management ability, revealing a more complex interaction between exercise and emotional health. Instead, screen media usage and other lifestyle factors play significant roles, underscoring the importance of a multifaceted approach. The role of smartphone usage in this chain mediation model suggests that reducing excessive reliance on smartphones has a potentially positive impact on improving emotion management ability. The results of this study not only provide new perspectives for educational institutions such as universities to formulate strategies to enhance students’ emotion regulation ability but also offer theoretical support for the development of effective intervention measures and strategies for promoting the health of college students. In addition, the findings emphasize the necessity of integrating emotional and digital literacy training into intervention programs, ensuring that students are equipped not only to manage emotions but also to use technology responsibly. It is recommended that universities optimize physical education and health teaching resources, cultivate exercise self-efficacy, improve e-health literacy, and provide appropriate guidance on smartphone use. This approach helps students develop a beneficial lifestyle for their physical and mental health, promotes their overall well-being, and equips them with comprehensive emotion management tools to face future challenges. Governments can also draw on these findings to construct policy frameworks that support healthy behaviors and promote moderate management of screen use.

## Data Availability

The raw data supporting the conclusions of this article will be made available by the authors, without undue reservation.
